# Assessment of human expertise and movement kinematics in first-person shooter games

**DOI:** 10.3389/fnhum.2022.979293

**Published:** 2022-11-29

**Authors:** Ian Donovan, Marcia A. Saul, Kevin DeSimone, Jennifer B. Listman, Wayne E. Mackey, David J. Heeger

**Affiliations:** Statespace Labs, Inc., New York, NY, United States

**Keywords:** visuomotor psychophysics, speed-accuracy tradeoff, motor acuity, movement kinematics, esports

## Abstract

In contrast to traditional professional sports, there are few standardized metrics in professional esports (competitive multiplayer video games) for assessing a player's skill and ability. We assessed the performance of professional-level players in Aim Lab^TM^, a first-person shooter training and assessment game, with two target-shooting tasks. These tasks differed primarily in target size: the task with large targets provided an incentive to be fast but imprecise and the task with large targets provided an incentive to be precise but slow. Each player's motor acuity was measured by characterizing the speed-accuracy trade-off in shot behavior: shot time (elapsed time for a player to shoot at a target) and shot spatial error (distance from center of a target). We also characterized the fine-grained kinematics of players' mouse movements. Our findings demonstrate that: 1) movement kinematics depended on task demands; 2) individual differences in motor acuity were significantly correlated with kinematics; and 3) performance, combined across the two target sizes, was poorly characterized by Fitts Law. Our approach to measuring motor acuity has widespread applications not only in esports assessment and training, but also in basic (motor psychophysics) and clinical (gamified rehabilitation) research.

## 1. Introduction

The principled study of digital game performance is in its infancy (Huang et al., 2017; Campbell et al., 2018; Listman et al., 2021), even though video games have been popular for decades (Gee, 2003; Kent, 2010; Egenfeldt-Nielsen et al., 2013; Ivory, 2015; Wolf, 2015). Performance in first-person shooter (FPS) video games relies on acquired perceptual and motor skills (Green and Bavelier, 2003) and evidence suggests that playing these games enhances visuomotor and cognitive skills (Green and Bavelier, 2008; Bavelier et al., 2012) in a variety of visual and cognitive tasks (Green and Bavelier, 2003, 2007; Dye et al., 2009; Colzato et al., 2013) in children and adolescents (Funk and Buchman, 1996; Adachi and Willoughby, 2013a,b,c; Chaarani et al., 2022) as well as adults (Green and Bavelier, 2006, 2007; Kowal et al., 2018). There is, consequently, much interest in research on this topic, including the potential applications in digital therapeutics (Hong et al., 2021), but there is a paucity of studies on gaming performance, itself.

Despite the rapidly growing popularity of competitive esports and the years spent by competitive and professional players to advance their skills (Popper, 2013), the field continues to lack established and objective benchmarks for individual skill. Many of the commonly used performance metrics in esports are unreliable measures of individual skill (Pedraza-Ramirez et al., 2020). Particularly in team games, key benchmarks (e.g., kill-death ratio, damage dealt, win-rate) confound the skill of an individual player with that of either their teammates, the opposing team, or the coordination of the team as a whole (Voida et al., 2010). For instance, a player with mediocre skill is still capable of obtaining a high rank simply by playing with individuals of a much higher skill level. The opposite is true for a great player with lesser-skilled teammates. Reliable, repeatable, and objective individual skill assessments with both intra- and inter-individual comparisons, on both short and longitudinal timescales, are needed to characterize true player rankings, efficacy of training, and to evaluate the impact of hardware, software, and player health (e.g., posture, exercise, sleep, diet, and dietary supplements) on performance.

Visuomotor psychophysics offers experimental approaches that are well-suited to assess individual players' FPS performance. Successful FPS play requires efficient identification and localization of relevant visual stimuli, and dynamic movements followed by well-timed shot responses (Pluss et al., 2020). One fundamental facet of FPS play is “flicking”—the action of rapidly moving the cross-hair to an object or enemy and firing a shot to damage or destroy the target. When playing with a computer mouse and keyboard, which is typical for expert players, flicking can be achieved by reach movements when targets are far away, or wrist and finger movements when targets are nearby. Protocols for measuring such ballistic kinematics are well established, and have been applied in a broad range of cognitive and visuomotor tasks (Desmurget and Grafton, 2000). As of yet, specific knowledge regarding FPS performance, based on rigorous visuomotor psychophysics models, remains scarce. Visuomotor skills are specialized, and are often constrained to the contexts and modalities in which they are learned (Fahle, 2005; Heuer and Hegele, 2008; Maniglia and Seitz, 2018). Thus, current laboratory-based visuomotor psychophysics research is not directly applicable to FPS performance.

Fitts Law has been shown to characterize visuomotor performance for a wide range of tasks (Fitts, 1954; Boritz et al., 1991; Soukoreff and MacKenzie, 2004; Zhai et al., 2004), including an FPS task (Looser et al., 2005). The law predicts that the time required to rapidly move to a target is a function of the ratio between the distance to the target and either the size of the target or the spatial error of the movement.

Motor acuity, complementary to Fitts Law, is defined as the ability to execute actions more precisely and within a shorter amount of time (Müller and Sternad, 2004; Shmuelof et al., 2012, 2014; McDougle and Taylor, 2019; Wilterson, 2021). There is a paucity of studies examining motor acuity, a gap likely linked to the tight resource constraints on laboratory-based studies. The handful of lab studies that examine motor acuity have used relatively simple motor tasks (Flatters et al., 2014), like drawing circles as fast as possible within a predefined boundary (Shmuelof et al., 2012), throwing darts (Martin et al., 1996), or center-out reaching and grasping (Jordan and Rumelhart, 1992; Shadmehr and Mussa-Ivaldi, 1994). Our group previously used a large sample of Aim Lab^TM^ performance data (over 7,000 players and over 60,000 repeats of the 60 s Gridshot task) over a period of months to examine motor learning (Listman et al., 2021), using hits per second as a proxy for motor acuity. Here, we propose a new approach for measuring motor acuity and calculating flicking skill by characterizing an individual player's speed-accuracy tradeoff, which we call the Flicking Skill Assessment (FSA).

It is both well established in the literature and widely discussed in the competitive gaming community that human performance exhibits a speed-accuracy tradeoff (SAT) (Heitz, 2014; Pluss et al., 2020): the speed of a response or action is negatively correlated with the accuracy or precision of that action. Players can be very fast and less accurate, very accurate and slow, or somewhere in between. This effect can be evident for different aspects of speed (e.g., reaction time, movement speed) and different aspects of accuracy (e.g., percent of correct responses/decisions, movement accuracy and variability). A hallmark of skillful SAT performance is the ability to adapt to current demands and prioritize speed and accuracy relative to each other. If a task requires very fast responses or movements, a player may sacrifice accuracy to maximize speed. If there is a high cost to incorrect responses, a person may take longer to respond or move more slowly to maximize accuracy. In FPS performance, one way to characterize SAT is in terms of shot behavior, with the spatial error of shots representing accuracy and shot time (elapsed time for a player to shoot at a target) representing speed. Performance on a single task with a single incentive for speed vs. accuracy is insufficient to estimate a player's motor acuity, as two players with the same skill may choose a different strategy in managing the SAT. Thus, skill cannot be characterized from a single data point, since skill and strategy would be confounded. The FSA assesses performance in a plurality of conditions in which priorities or incentives for speed and accuracy differ, to isolate an individual player's skill from their chosen trade-off between speed and accuracy. The result of this assessment is a measure of motor acuity for quantifying skill in FPS flicking tasks, which is independent of bias or strategy.

We used Aim Lab^TM^, an FPS video game that assesses and trains players to optimize their performance, i.e., an automated personal trainer, to collect data. Aim Lab^TM^ comprises a wide range of tasks that replicate gaming scenarios, each matched with a particular data analysis procedure and inspired by visuomotor psychophysics. We recruited professional esports athletes (specializing in several different game titles and roles within a team) to play two tasks, one of which incentivized speed and the other of which incentivized precision. In addition to assessing SAT and motor acuity via shot behavior, we also characterized kinematics of players' mouse movements. Players often aim for and destroy a target by initiating a movement toward the target, increasing and subsequently decreasing movement speed, then firing a shot after slowing down or coming to a full stop. We characterized these movements by fitting a sigmoid to the time-series of mouse positions, with the best-fit parameter values indicating a movement's kinematics (i.e., reaction time to initiate a movement, movement speed, movement accuracy, variability or precision of a plurality of movements). In addition to these typical flick-and-land movements, players may choose to maximize speed by shooting “on the fly” instead of slowing down before firing. FPS players refer to this type of movement behavior as a “swipe” movement, and we developed an approach to characterize the extent to which movements resembled a swipe vs. a typical flick-and-land movement.

The aim of this study was to determine if movement kinematics depends on task demands, individual differences in motor acuity are correlated with individual differences in movement kinematics, and if FPS performance is well characterized by Fitts Law. We found that: 1) movement kinematics depended on task demands (reaction times were shorter, movement speed was faster, and movements were more “swipey” for the task that incentivized speed over precision); 2) individual differences in motor acuity were significantly correlated with kinematics; and 3) performance, combined across the two target sizes, was poorly characterized by Fitts Law.

The FSA, our method for measuring motor acuity, may be used to assess and compare skill between and within players, training to improve skill, and tracking change in performance over time. The flexibility, accessibility, and engaging nature of FPS games also makes the FSA a promising tool for other applications, such as motor rehabilitation and monitoring and training cognitive health and fitness.

## 2. Methods

### 2.1. Participants

Performance data from 32 professional and semi-professional male esports players (mean age = 22.47 ± 3.62) were collected, specializing in different FPS game titles: 4 Valorant players (Riot Games, 2020), 10 PUBG: Battlegrounds players (PUBG Studios, 2017), and 18 Rainbow Six Siege players (Ubisoft, 2015). Data were acquired initially for commercial purposes, stored separately from player account data and without personal identifiers. For this study, the data were reanalyzed *post-hoc*, thus informed consent was not required (Advarra Institutional Review Board).

### 2.2. Apparatus

Aim Lab^TM^ is a commercial software product written in the C# programming language using Unity game engine (Helgason et al., 2005). Unity is a cross-platform video game engine used for developing digital games for computers, mobile devices, and gaming consoles (Brookes et al., 2020). Players download Aim Lab^TM^ directly to their desktop or laptop PC. Players control their virtual weapon in Aim Lab^TM^ tasks using a mouse and keyboard, while viewing the game on a computer screen.

The participants each completed the tasks remotely using their own gaming set-up. This included their own hardware such as the PC, monitor, mouse and mouse pad. In addition, there were other individual settings such as display size, viewing distance, chair height, and mouse counts per inch (CPI). We assumed that there was a wide range of equipment combinations amongst the participants, and we did not control for any differences in gaming set-ups between players other than CPI and mouse sensitivity (see Data Analysis, below); however, mouse acceleration was disabled. The orientation of the player's view of the environment (controlled by the player's mouse) was recorded in Euler angles and sampled at 120 Hz, then were uploaded to our secure servers. The crosshair, marked with a dot, was placed always at the center of the screen and corresponded to the direction in which a shot would be fired. When the player clicked the left mouse button to shoot, the projectile would move in a straight line pointing away from the player's virtual avatar.

### 2.3. Task descriptions

Aim Lab^TM^ includes a variety of different task scenarios for skill assessment and training, each tailored to a facet of FPS play. These task scenarios assess and train a number of psychophysical processes, including: visual detection, motor control, tracking moving targets, auditory spatial-localization, change detection, working memory capacity, cognitive control, divided attention, and decision making. Each task can be customized to prioritize accuracy, speed, or any basic component of performance over others. During every round, players are granted points for each target that they successfully track or shoot and destroy. Additional points are rewarded for targets destroyed more quickly or tracked for a longer period. Players attempt to maximize their score on each round by destroying or tracking as many targets as possible.

In this study, we used task scenarios that assessed the players' flicking skill, a combination of visual detection, motor planning, and motor execution. Specifically, we used two tasks, Gridshot and Sixshot, that were very similar but differed in target size to characterize each player's SAT and estimate their motor acuity. The tasks were designed to incentivize players to maximize their score by either prioritizing accuracy over speed when targets were smaller, or prioritizing speed over accuracy when targets were larger. Each of the 32 professional-level esports athletes played 6 runs of each of the two tasks, after previous sessions to familiarize themselves with the tasks.

#### 2.3.1. Gridshot

**Figure 1:**
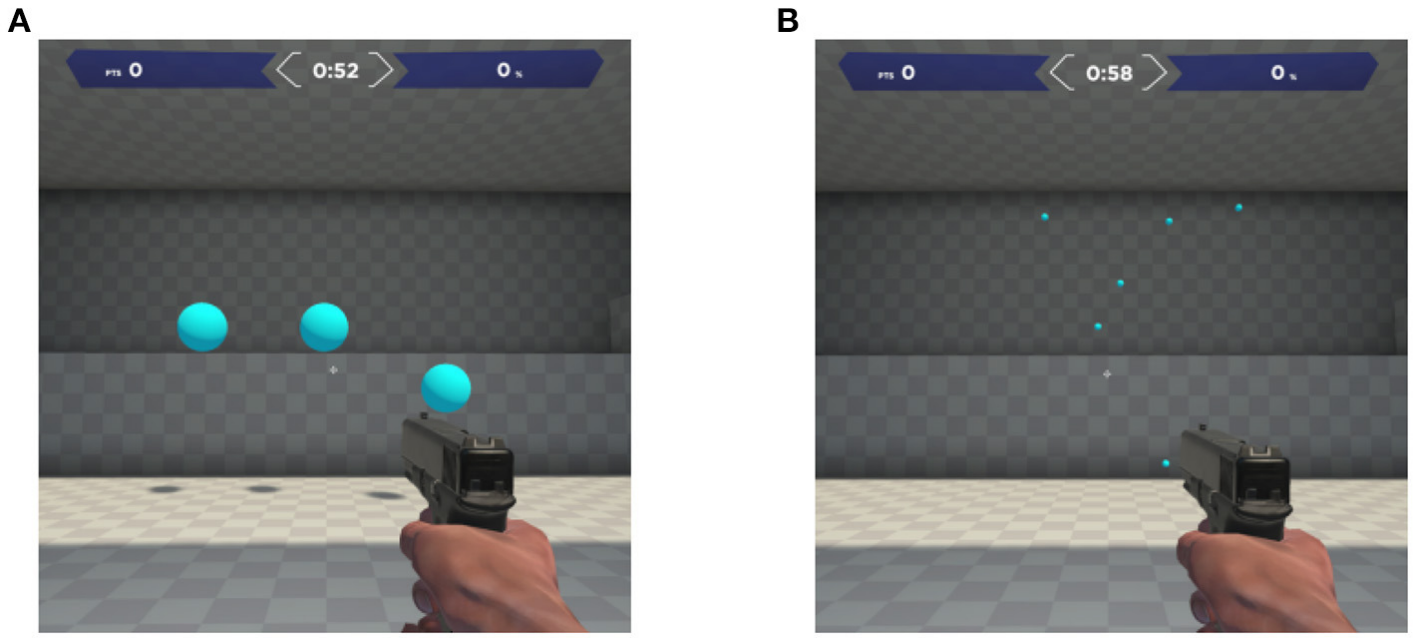
Screenshots from Aim Lab^TM^, displaying a single frame from the: **(A)** Gridshot and **(B)** Sixshot tasks.

In Gridshot (Figure 1A) there are 3 targets presented simultaneously at any given time, with a new target appearing (spawning) once an existing target is destroyed. There is neither a ceiling nor floor effect, due to this self-paced design of the task. All targets are the same size, ranging between 1.3° and 1.7° (degree of visual angle), assuming a range of viewing distances and a range of values for the field of view in the virtual environment of the game (set by the player). Spawn locations are randomized to 1 of 25 positions in a 5 × 5 grid, ranging between 4.8° and 9.1° wide and 5.1° and 7.8° high and similarly depending on viewing distance and field of view. The player destroys a target by moving their mouse to aim then clicking the left mouse button to shoot. Due to multiple targets being present at once, and combined with the unlimited target duration and no explicit incentive to destroy any specific target, the players, themselves, must decide the order in which to destroy the targets. Players receive immediate feedback upon target destruction; the game emits an explosion sound and the orb-shaped target splinters into multiple pieces which then disappear. Players receive a summary of performance feedback after each 60 s run of Gridshot. These summary metrics include score, hits per second (number of targets successfully destroyed per second), and hit rate (percentage of shot attempts that successfully hit a target). Points are added to the score when the targets are hit and, in contrast, points are subtracted for shots that missed the target. The player's score is displayed at the top of the screen throughout the run, automatically calculated in the game's software, and sent to a secure server. The number of points added for each target destroyed is scaled by the time since the previously destroyed target. In other words, the shorter the time it takes to destroy the next target relative to when the previous target was destroyed, the more points are added to the player's score. Thus, players are incentivized to quickly plan their next movement and shoot targets rapidly. Even though players are shown multiple metrics at the end of each run, it is likely that they are consciously optimizing for increased score during the actual runtime of the task.

#### 2.3.2. Sixshot

Sixshot (Figure 1B) is very similar to Gridshot, whereby they share identical possible target locations. However, the Sixshot task bears the following differences to Gridshot: 6 targets are present at a time rather than 3, and the targets are approximately 14% the size of Gridshot targets. If optimizing for score, the smaller target size demands much greater shot accuracy from the player than the targets in Gridshot.

## 3. Data analysis

### 3.1. Flicking skill assessment and fitts law

The Flicking Skill Assessment (FSA) was based on measuring shot time (time interval between target appearance and the subsequent shot) and shot spatial error (distance from center of a target). Specifically, we characterized the SAT between the two tasks by plotting shot speed (1/median shot time) vs. shot precision (1/median shot spatial error). The same two measures (shot time and shot spatial error) were used to assess Fitts Law.

#### 3.1.1. Mouse calibration

To measure the player's physical mouse movements, we converted the player's orientation in the virtual environment to the corresponding movements of the mouse on the mouse pad, which has units of centimeters (cm). This required additional information about the relationship between physical mouse movements and changes in player orientation, which varied across players due to their settings in Aim Lab^TM^, and their own hardware and software. For each player, we recorded their in-game settings that governed the field of view and their mouse sensitivity—the magnitude of a change in camera rotation (in degrees,°) derived from a single increment or count of the mouse hardware. The individual mouse sensitivities were constant across x and y for each player. Each player's mouse (either hardware, software, or both) had a unique setting that determined the number of counts that resulted for one inch of distance traveled (counts per inch, CPI).

Player camera orientation was converted to physical mouse movements as detailed below:

1. Mouse sensitivity × 0.05 = angle increment (degrees turned per count)
2. Total degrees turned/angle increment = counts
3. (Counts/CPI) × 2.54 = physical distance traveled (cm)

The value of 0.05 is an arbitrary constant used in the Unity software code to scale mouse counts to degree increments. After this conversion, across all players and tasks the maximum target distance was 5.60 cm. Thus, all targets were proximal enough that players could have landed on every target using a combination of only wrist rotations and bending or straightening the fingers, i.e., forearm or elbow movement were not strictly necessary.

#### 3.1.2. Movement parsing

To assess movement kinematics, we first parsed the time-series of each player's orientation in the virtual environment of the game, as controlled by their mouse movements. Each time-point (or sample) was labeled as either in-motion or stationary. Epochs were labeled as in-motion after several consecutive samples exceeded a velocity threshold. Conversely, epochs were labeled as stationary after several consecutive samples fell below a velocity threshold. The number of consecutive samples used and the values of the thresholds were determined using a proprietary algorithm, procured from a large dataset of Aim Lab^TM^ players.

The primary movement for each target was identified as the largest amplitude movement in the direction of the target. During each 60-s run of the tasks, these primary movements were often followed by a corrective movement, i.e., an action in response to not having destroyed the target with the primary movement. For instance, the primary movement could be hypermetric, indicating that the player's crosshair passes beyond the target and they must initiate a corrective movement in the opposite direction back toward the target. Alternatively, the primary movement could instead be hypometric, falling short of the target and requiring a corrective movement in the same direction as the primary movement. At times, multiple corrective movements were needed to destroy the target. These component movement were distinguished from one another by the movement parsing procedure outlined above. Each component movement was fit separately, whether it was a primary or corrective movement.

#### 3.1.3. Movement kinematics

**Figure 2:**
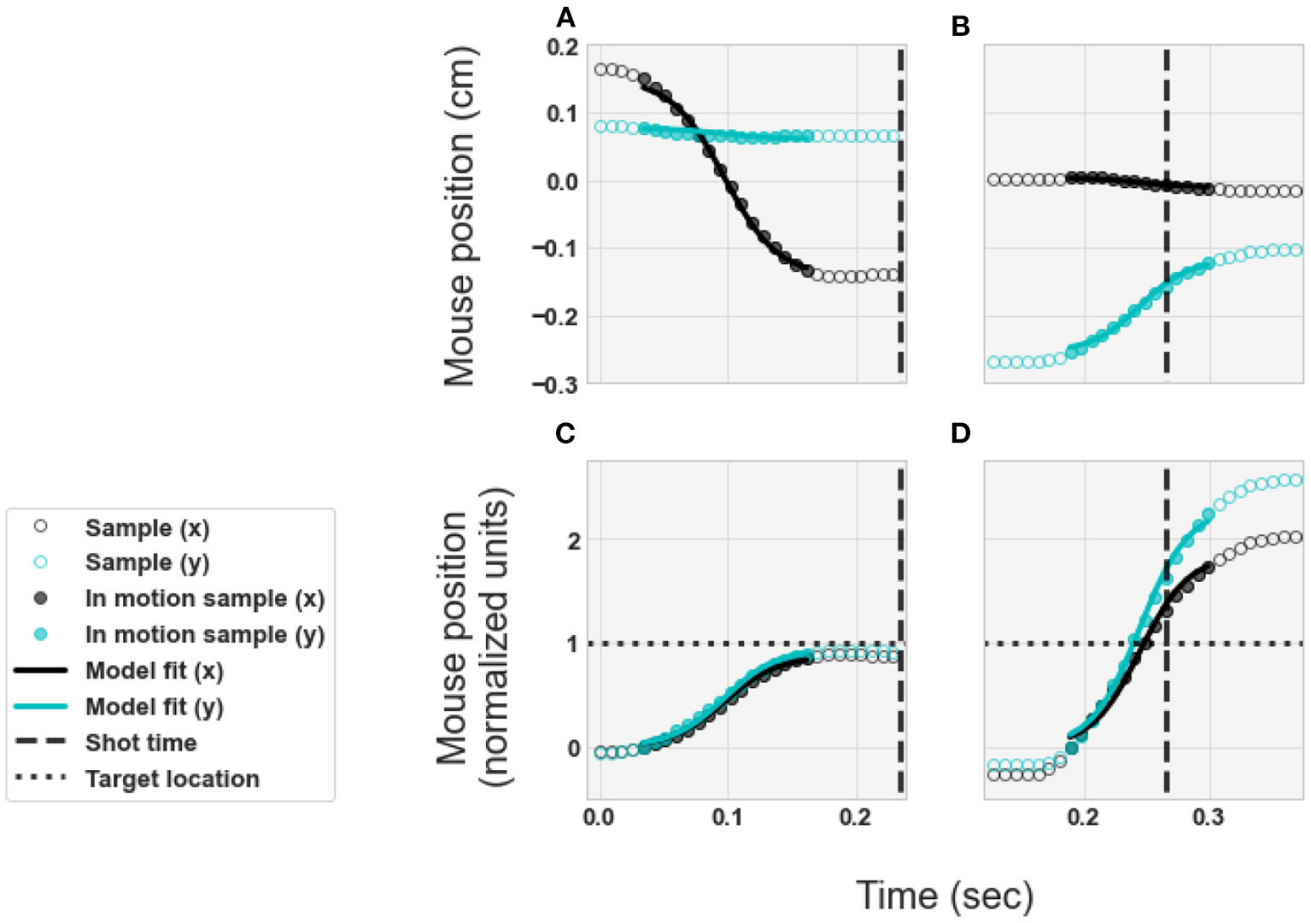
Examples of the movement trajectories and model fits. **(A,C)** Example flick-and-land. **(B,D)** Example swipe. **(A,B)** Physical movements in units of centimeters. **(C,D)** Normalized units - so that 1 corresponds to target location. Circles illustrate the samples of the player's movement trajectory. Curves illustrate the models of the movement trajectories, as expressed by Equations (1–3), with best-fit values for the parameters.

By fitting parametric functions to the mouse movement data, we quantified the features of an individual's motor behavior. This resulted in measurements of speed, precision, accuracy, reaction time (henceforth shortened to SPAR), as well as swipiness (see Section 3.5). Specifically, player orientation during each period labeled as in-motion was fit with a sigmoidal function:


(1) $$\begin{array}{l} f\left(t;a,b,c\right)=\frac{a}{1+e^{b}\left(t-c\right)} \end{array}$$

(2) $$\begin{array}{l} x\left(t\right)=f\left(t;p_{1},p_{3},p_{4}\right) \end{array}$$

(3) $$\begin{array}{l} y\left(t\right)=f\left(t;p_{2},p_{3},p_{4}\right) \end{array}$$

where Equation (1) defines the sigmoid. The values of *x(t)* in Equation (2) represent a model of the horizontal component (rotation about the y-axis) of the movement trajectory for each time-point sample. The values of *y(t)* in Equation (3) represent a model of the vertical component (rotation about the x-axis) of the movement trajectory for each time-point sample. Example illustrations of the model components *x(t)* and *y(t)* are shown in Figure 2. The top row of Figure 2 shows two example movements in units of centimeters, transformed using the mouse calibration. The bottom row of Figure 2 shows the same two example movements, scaled to normalized units so that 1 corresponds to the target location. The left column of Figure 2 shows an example of a flick-and-land. In these panels, the shot (vertical dashed line) occurs after the movement ends and the movement lands at the target location (horizontal dotted line). The right column of Figure 2 shows an example of a swipe. The shot (vertical dashed line) is made during the middle of the movement, and the movement lands well past the target location (horizontal dotted line). The values of the parameters (*p*_1_, *p*_2_, *p*_3_, *p*_4_) were fit to each individual movement trajectory using the Levenberg-Marquardt algorithm (Levenberg, 1944). In Equations (2) and (3), the x- and y- movement components were fit with shared parameters *p*_3_ and *p*_4_.

SPAR metrics were then calculated from the best-fit parameter values:

1. Speed (cm/second): peak speed at the midpoint of the movement.
2. Precision (1/median absolute % distance to the target): variability in the accuracy across trials/targets.
3. Accuracy (% distance to the target): distance between the landing location of the movement and the center of the target.
4. Reaction time (s): time interval between when the target appeared and the initiation of the movement, i.e., when the movement reached 5% of its endpoint.

Specifically, the movement speed and accuracy of each parsed movement were quantified as:


(4) $$\begin{array}{l} Speed=\frac{a_{m}}{f^{\prime }\left(p_{4};a_{m},p_{3},p_{4}\right)} \end{array}$$

(5) $$\text{Accuracy}=\frac{e^{T}u}{a_{t}}$$

(6) $$f^{\prime }(t;a,b,c)=bc[f(t;1,b,c][1-f(t,1,b,c)]$$

(7) $$\begin{array}{l} a_{m}=\sqrt{{\left(p_{1}\right)}^{2}+{\left(p_{2}\right)}^{2}} \end{array}$$

(8) $$\begin{array}{l} a_{t}=\sqrt{{\left(x_{t}\right)}^{2}+{\left(y_{t}\right)}^{2}} \end{array}$$

(9) $$e=(p_{1}-x_{t},p_{2}-y_{t})$$

(10) $$\begin{array}{l} u=\frac{\left(x_{t},y_{t}\right)}{∥\left(x_{t},y_{t}\right)∥} \end{array}$$

The function *f'(t)* is the derivative of the sigmoidal function (Equation 6). The value of *a*_*m*_ represents the amplitude of the movement (Equation 7) and the value of *a*_*t*_ represents the distance to the target location (Equation 8). The vector **e** represents the movement error (Equation 9) and the vector *u* represents a unit vector in the direction of the target location (Equation 10). Movement speed was re-scaled using the mouse calibration protocol (to have units of: cm/second, see Section 2.4.1). Precision was calculated as the variability in accuracy, using a robust measure of variability (the median of the absolute difference from the target center, rather than the standard deviation) to minimize the impact of outliers.

#### 3.1.4. Swipiness

To characterize the degree to which each ballistic movement resembled a swipe (the action of shooting on the fly) vs. a flick-and-land (slowing down and stopping before firing) movement, we compared the time of each shot that a player made with the time of the midpoint from the associated ballistic movement. An ideal swipe corresponds to firing a shot at the midpoint of a movement, i.e., the time point with maximum speed. On the other hand, an ideal flick-and-land corresponds firing a shot only after the movement has ended, long after the midpoint of the movement. Thus, swipiness was calculated from dividing the time of the shot by the time of the midpoint of the movement (*p*_4_), divided by 2. This resulted in a swipiness value of 0.5 (arbitrary units) for an ideal swipe, and a swipiness value ≥ 1 for an ideal flick-and-land. There was no swipiness value computed for a movement that had no associated shot, i.e., if there was no shot between the initiation of one movement and the initiation of the next movement.

Swipiness itself is a granular measure of shot speed as it relates to the movement trajectory, with lower swipiness indicating that the firing of a shot occured earlier in the trajectory. Additionally, the absence of a swipiness value indicates that a movement was not associated with a shot. Across trials within a certain context or task, the number of movements with no swipiness value reflects the need for players to make multiple movements to destroy targets.

### 3.2. Data post-processing

For each player, we computed SPAR for every movement in Gridshot and Sixshot and swipiness for every movement that had a corresponding shot fired. We then applied several steps of post-processing.

Firstly, upper and lower band thresholds were applied to remove outliers (presumed to be failures in the trial parsing or SPAR fits). For all of the metrics, the 95th percentile was used for the upper band value and 0 was used for the lower band value. Sigmoid fits with an r-squared value of less than 0.5 were pruned. Each accuracy value was multiplied by 100, to convert from proportion to percent of the distance to the target. We then subtracted 100 from each value such that hypometric movements had negative values and hypermetric had positive values, i.e., accuracy values of 0 indicate that the player fired a shot directly at the center of the target to destroy it. We computed the median of each metric, separately for each individual player, and separately for each task and movement-type condition. This yielded a total of 20 variables per player: 5 metrics (median speed, precision, accuracy, reaction time, and swipiness) × 2 movement types (primary vs. corrective) × 2 tasks (Gridshot vs. Sixshot).

**Figure 3:**
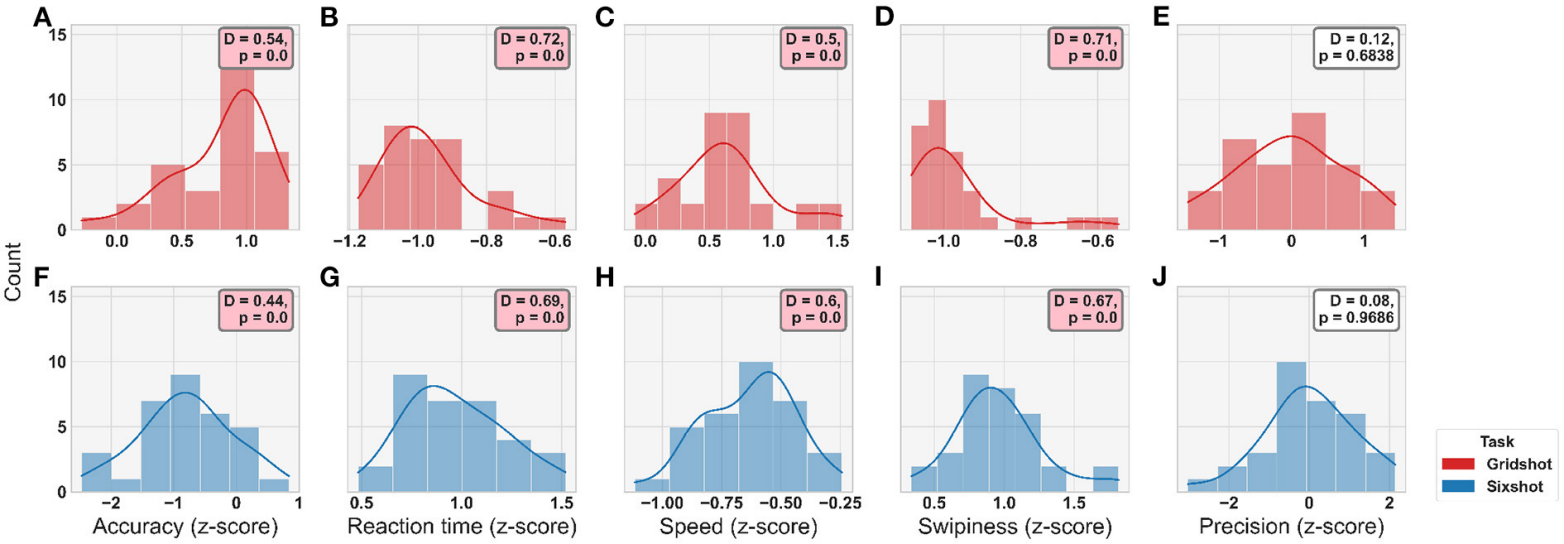
Distribution plots for each metric by task (see Legend). **(A–E)** Gridshot. **(F–J)** Sixshot. Statistical results from Kolmogorov-Smirnov test for normality displayed in annotation boxes; where D, KS-statistic and P, *p*-value. Pink annotation boxes indicate a *p* < 0.05.

A z-score was calculated for each metric across players, combining both Gridshot and Sixshot (for each metric and movement type) to permit statistical comparisons between the tasks. The distributions of these data are shown in Figure 3. Once z-scored, data was tested for normality using Kolmogorov-Smirnov; the resulting *p*-values indicated that the majority of metric distributions were non-normal (8 out of 10 variables where *p*s < 0.05) and therefore non-parametric approaches to statistical analysis were taken. To mitigate the confounding effects of mouse sensitivity, this vector was converted into a diagonal matrix and regressed out of both the movement kinematics and motor acuity. To do so, we expressed the z-scored movement kinematic metrics **Y** (a 32 × 20 matrix: 32 players and 20 kinematic variables, as stated above) as a linear prediction of the z-scored mouse sensitivities **A** (a 32 × 32 diagonal matrix) multiplied by regression coefficients **X**:


(11) Y=AX

First, the inverse of the mouse sensitivity diagonal matrix **A**^#^ was calculated and multiplied by the movement kinematics matrix **Y** to estimate the regression coefficients **X^**:


(12) $$\hat{X}=A^{\#}Y$$

Second, we computed the components of the movement kinematics that could be predicted by the mouse sensitivities **Ŷ** by multiplying **X^** with the mouse sensitivity diagonal matrix **A**:


(13) $$\hat{Y}=A\hat{X}$$

Third, the residual movement kinematics matrix, **R**, was computed by subtracting **Ŷ** from **Y**:


(14) $$R=Y-\hat{Y}$$

Mouse sensitivity was correlated with motor acuity and with movement kinematics, but this correlation was removed by the regression procedure (Supplementary Figures S8–S10).

## 4. Results

### 4.1. Motor acuity

We predicted that pro and semi-pro players' would exhibit a SAT as a function of the target size. In particular, we predicted that players would exhibit systematic differences in movement behavior between Gridshot and Sixshot. Gridshot's large target size incentivizes players to maximize score by moving as quickly as possible with relatively low shot precision (1/median shot error, as measured by the distance to the center of the nearest target in cm). In comparison, Sixshot targets are much smaller and require high precision, which consequently means that players are motivated to slow down in order to maximum their score.

**Figure 4:**
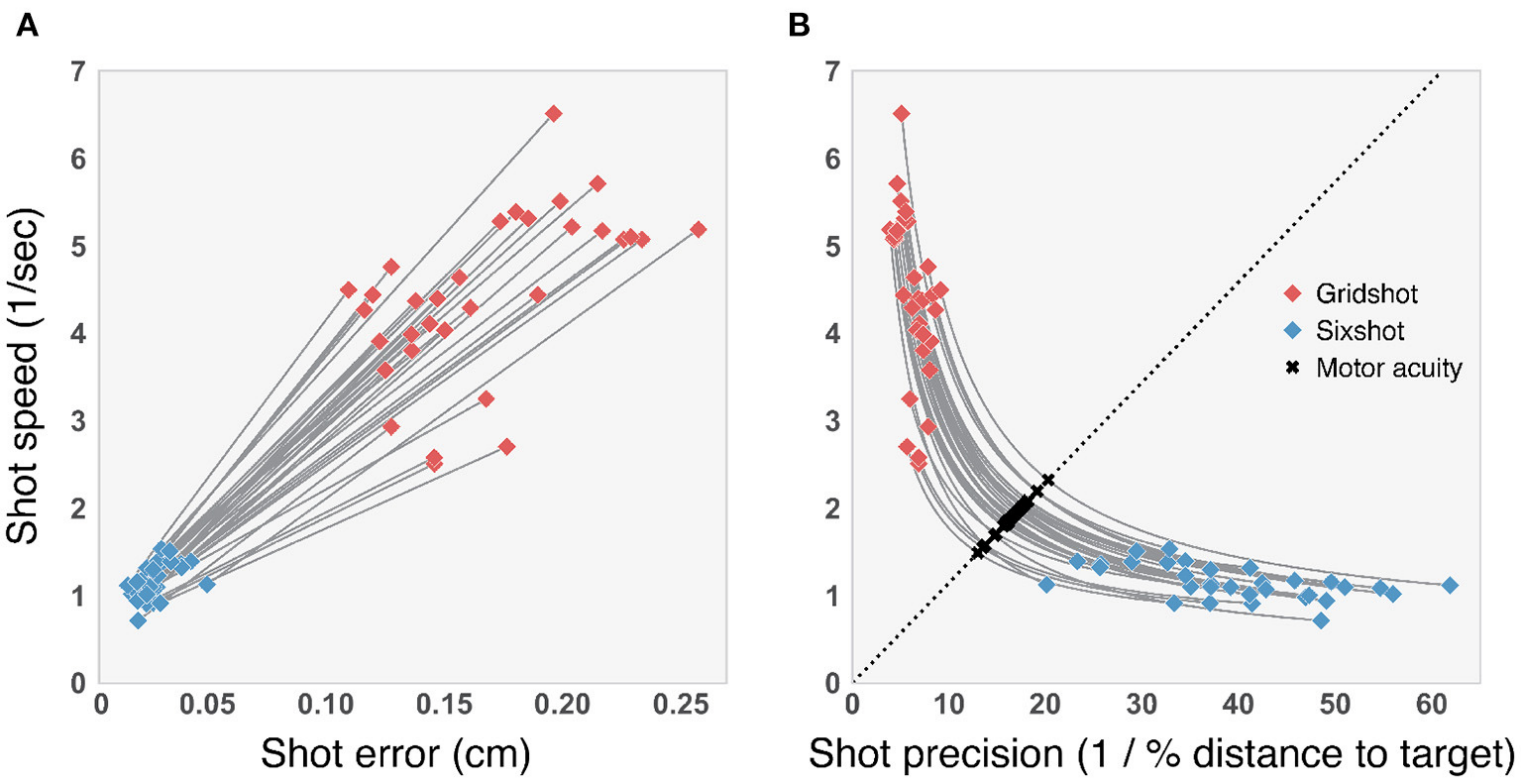
**(A)** Shot speed vs. shot error. Solid lines illustrate the line of best fit for each player by task (see Legend). **(B)** SAT curves. Dashed line spans from (0, 0) to three-times the standard deviation, plus the mean of the distribution of shot precision (x-axis) and shots speed (y-axis). The intersection of individual curves with the diagonal, marked with “ × ,” indicates motor acuity (arbitrary units).

Figure 4 illustrates the SAT and the Flicking Skill Assessment (FSA). The left panel of Figure 4 shows shot speed (1/median shot time) as a function of the median shot error for Gridshot and Sixshot. Each line connects the Gridshot and Sixshot data for a single player. Our findings indicate that each player followed the expected pattern in behavior: faster and more variable in Gridshot compared to Sixshot. This demonstrates that players responded strategically and appropriately to the different demands of the two tasks. The right panel of Figure 4 re-plots these results in terms of shot precision (1/median shot error). The curves represent the transformed lines from the left panel.

The dashed diagonal line spans from (0, 0) to three-times the standard deviation, plus the mean of the shot precision distribution (x-axis) and shots per second distribution (y-axis). This line represents the axis of motor acuity; SAT curves placed toward the upper right indicate better performance, i.e., higher precision combined with faster speed. We obtained a flicking skill value for each player, by identifying the point along the diagonal line that intersects with that player's SAT curve. Notably, these flicking skill values are related to certain aspects of movement kinematics, as detailed in the following subsections.

### 4.2. Fitts law

**Figure 5:**
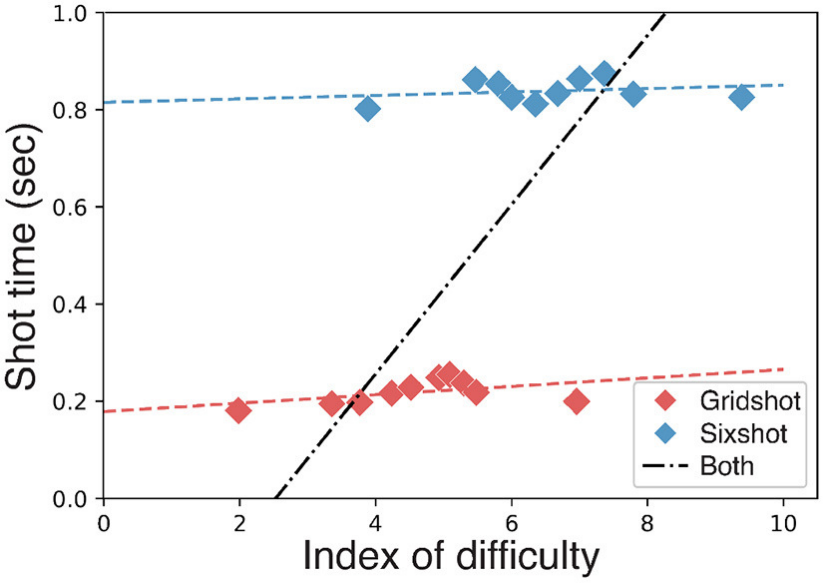
Fitts Law. Shot time as a function of target distance. Index of difficulty: log_2_(2*d*/*e*) where *d* is target distance and *e* is shot error. Blue line, regression line fit to Sixshot data. Red line, regression line fit to Gridshot data. Black line, failure to fit data from both tasks combined.

Performance, combined across the two target sizes, was poorly characterized by Fitts Law (Figure 5). For each target we measured the shot time (elapsed time between the destruction of a target and the first shot at the subsequent target), target distance (angular distance to the target when it first appeared) and shot error (angular distance between the shot and the center of the target). The logarithm of the ratio of target distance and shot error was computed, separately for each target, and then binned by decile. For each bin, we computed: 1) the median shot time; and 2) the median logarithm of the ratio of target distance and shot error. Fitts Law provided a good characterization of behavioral performance when fit separately to the data from each task (Figure 5, red and blue lines), but a very poor characterization of the data when combined across the two tasks (Figure 5, black line). In other words, shot times were well predicted by Fitts Law as a function of target distance and target error for each target size separately, but not for both target sizes together.

### 4.3. Task-dependence of movement kinematics

**Figure 6:**
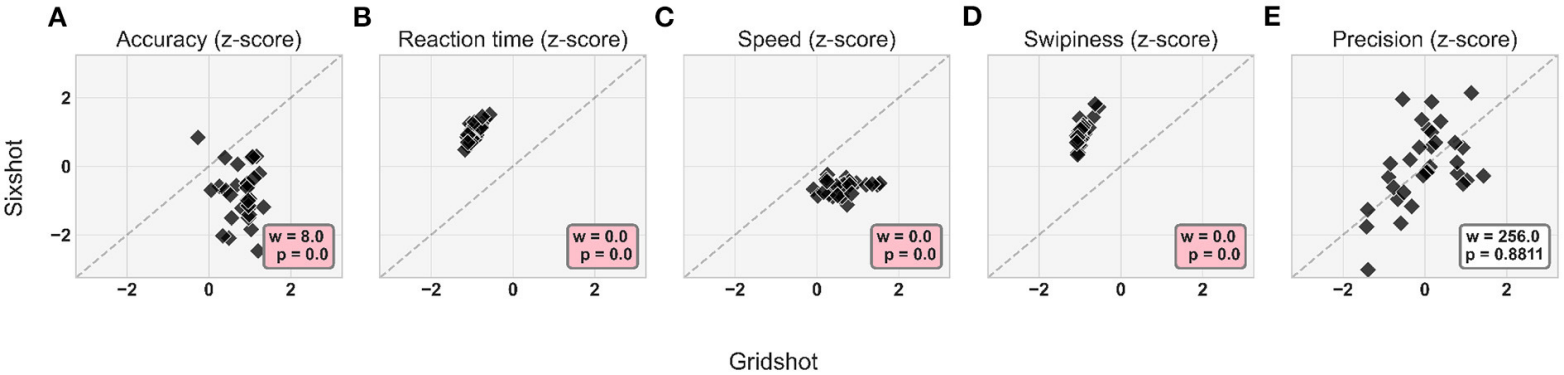
Differences in movement kinematics for Sixshot vs. Gridshot: **(A)** Accuracy. **(B)** Reaction time. **(C)** Speed. **(D)** Swipiness. **(E)** Precision. Each data point within each panel corresponds to a different player. Statistical results from the paired Wilcoxon signed-rank test displayed in annotation boxes; where w is sum of ranks, and p is *p*-value. Pink annotation boxes indicate a *p* < 0.05. The dashed diagonal represents the values at which the kinematic z-scores for Gridshot is equal to Sixshot.

Movement kinematics differed between the two tasks; Figure 6 illustrates each player's pattern of strategy change for each movement kinematic metric (after regressing out mouse sensitivity), with Gridshot on the x-axis and Sixshot on the y-axis. The results indicate that players were more hypometric in Sixshot compared to Gridshot (Accuracy; *p* < 1e-4). Players were faster to react (Reaction time; *p* < 1e-4), they moved their mouse at a faster pace (Speed; *p* < 1e-4), and fired earlier in their movement trajectory (Swipiness; *p* < 1e-4) in Gridshot compared to Sixshot. There was no statistical evidence of a significant difference in the variability of accuracy between tasks (Precision; *p* = 0.88), indicating that players were similarly consistent in their landing positions for Gridshot and Sixshot.

### 4.4. Individual differences in motor acuity and kinematics

**Figure 7:**
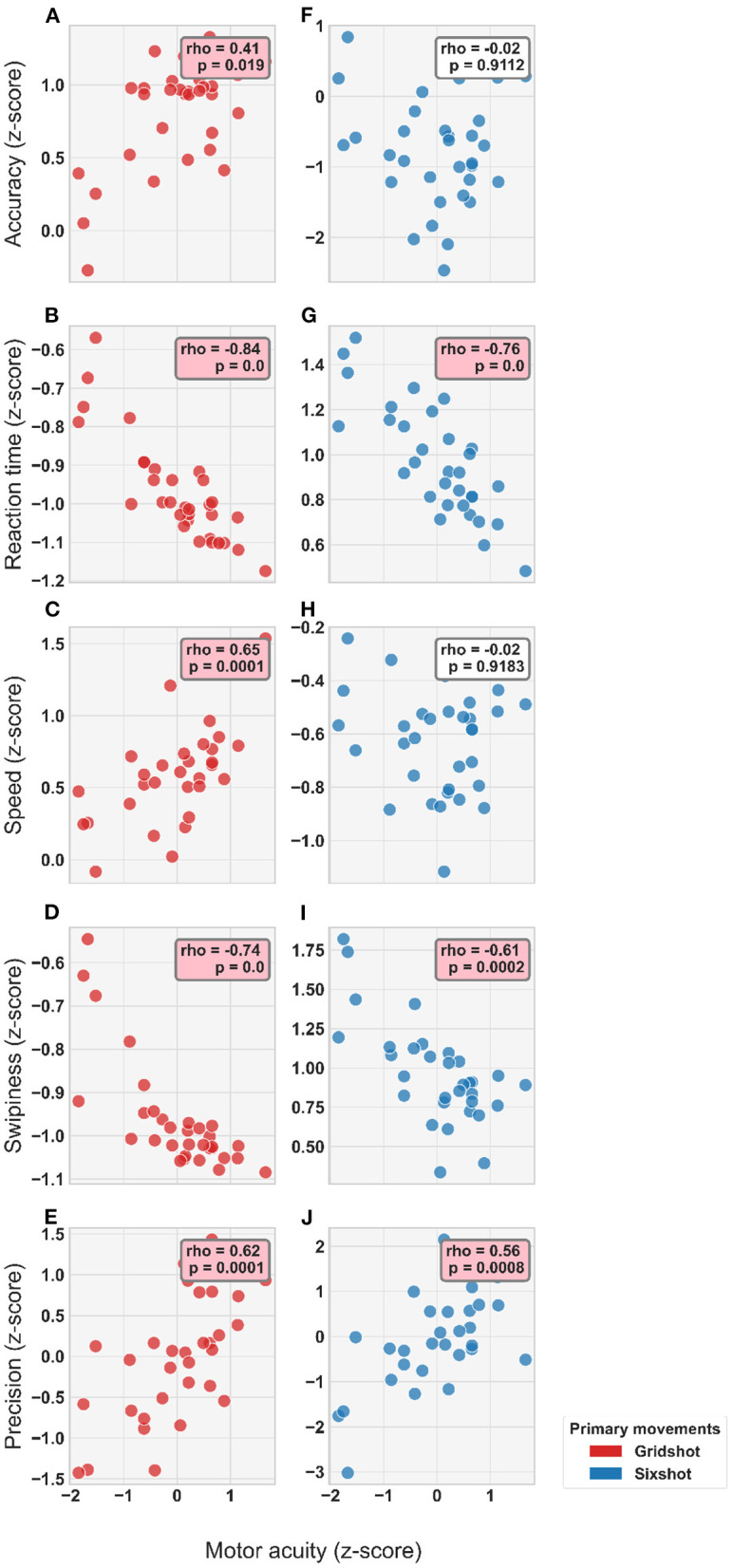
Correlation between player kinematics and motor acuity for each metric in each task (see Legend): **(A–E)** Gridshot. **(F–J)** Sixshot. Statistical results from Spearman's Rank Correlation displayed in annotation boxes; where rho, Spearman's Correlation Coefficient and P, *p*-value. Pink annotation boxes indicate a *p* < 0.05.

Motor acuity, as measured with the FSA, was predictive of individual differences in movement kinematics. Figure 7 shows the relationship between individual differences in motor acuity and movement kinematics (after regressing out mouse sensitivity). For Gridshot, motor acuity was negatively correlated with reaction time (*p* < 1e-4) and swipiness (*p* < 1e-4), while positively correlating with accuracy (*p* = 0.019), speed (*p* = 1e-04), and precision (*p* = 1e-04). For Sixshot, motor acuity was also negatively correlated with reaction time (*p* < 1e-4) and swipiness (*p* = 2e-4), and positively with precision (*p* = 8e-4). For both tasks, the interpretation of these results is that players with greater motor acuity initiated movements more quickly, landed with lower variability, and fired shots earlier in the movement trajectory. Moreover, in Gridshot only, players with greater motor acuity exhibited faster movement speed and better (less hypometric) accuracy.

## 5. Discussion and conclusion

In this study, we developed and validated the flicking skill assessment (FSA), a novel approach to objectively assess individual player skill in FPS games. Furthermore, we elucidated the systematic relationship between motor acuity (measured by characterizing speed-accuracy tradeoff, SAT) and movement kinematics. Our results reveal the individual differences in motor acuity and movement kinematics between professional-level FPS players, as well as differences in kinematics across tasks with different target sizes. The proposed FSA is an elegant and efficient approach which requires only an adjustment of target size between similar tasks.

The players' performance were found to differ between tasks, whereby players used a more conservative strategy in Sixshot compared to Gridshot. The movement kinematics provided detailed information about the nature of this shift in strategy between tasks: players take longer to plan their movements, move slower, fire later in the trajectory, and are more likely to land hypometrically in Sixshot compared to Gridshot. Given that the targets in Sixshot are small and require less error compared to large targets in Gridshot, this adaption in shot and movement behavior is a rational and efficient approach. Players sacrifice speed for accuracy and display more of a bias toward conservative (hypometric) landing positions during Sixshot compared to Gridshot.

Our analysis characterized individual differences in motor acuity and movement kinematics across professional-level esports athletes. Players differ in the degree to which they can be both fast and accurate. Players with a higher motor acuity value, i.e., their SAT curves are further upwards and rightwards, were both faster and more accurate overall than players with a lower motor acuity value, and are thus considered to have a higher skill level. Critically, motor acuity significantly correlated with a subset of movement kinematics. This demonstrates that individual differences in flicking skill are predictive of individual differences in kinematics. Greater motor acuity accompanied faster reaction times, greater precision and a tendency to shoot earlier in the trajectory (swipiness) for both Gridshot and Sixshot (Figure 7).

Perhaps surprisingly, Fitts Law provided a poor characterization of performance when combined across the two tasks (i.e., across the two target sizes). Fitts Law provided a good characterization of behavioral performance when fit separately to the data from each task, but a very poor characterization of the data when combined across the two tasks. We speculate that this failure of Fitts Law reflects a difference in strategy between the two tasks, specifically, that players tended to be more swipey for Gridshot than for Sixshot. A previous study found excellent agreement between Fitts Law and behavior in an FPS task (Looser et al., 2005), but the participants in that study were either amateur gamers or did not report playing FPS games at all. Swiping is an advanced skill that is commonly used by highly competitive and professional players when speed is of paramount importance.

One crucial design element of the FSA is the inclusion of more than one task, each with distinct incentives regarding speed and precision. The placement of an individual's entire SAT curve is the key to quantifying motor acuity, rather than the placement of a single data point. The SAT curve itself represents the possible combinations of shot speed and shot precision given the player's skill, with the player's current strategy determining where along the curve their current speed and precision is drawn from. Two players with identical SAT curves could have different strategies within the same task, such that their performance would sample different values for speed and precision on the same curve. Including multiple tasks, each with distinct incentives for speed and precision, enables the characterization of SAT across possible incentives, and thus isolates motor acuity.

Indeed, we have now implemented a version of the FSA with 3 different target sizes (Supplementary Figure S11). In this task, called Adaptive Reflexshot, one target at a time appears in a randomized location, confined to an imaginary ellipse in front of the player's virtual avatar. Players have a limited time to destroy each target before it “times-out” and disappears. To provide a visual cue of time remaining, each target gradually becomes more transparent and then disappears. The target presentation duration is titrated according to performance: the duration is decreased (it becomes transparent more quickly) on the next trial after a target is destroyed and the duration is increased (it becomes transparent more slowly) on the next trial after a target time-out, separately for each target size.

A second crucial design element is the measurement of both speed and precision. It is not possible to use a single performance metric as a proxy for motor acuity, considering it is defined by both speed and accuracy (Shmuelof et al., 2012). For instance, retrieving only shot speed from both tasks can be confounded by players simply shooting sooner with no ramifications for being inaccurate—a behavior that does not reflect high skill.

Our measure of motor acuity and the movement kinematics were derived from different behavioral sources: shot behavior and mouse movements, respectively. By identifying a systematic relationship between the two sources, our findings demonstrate that individual differences in player skill correspond to individual differences in underlying movement kinematics. We propose that the FSA, a measure of motor acuity, represents a powerful tool for quantifying skill in FPS flicking tasks. One of the major benefits of the FSA is the capability to isolate player skill from context-dependent strategy regarding SAT. Indeed, a player can achieve their best possible skill ranking by matching shot behavior with task incentives. However, their underlying ability will determine the upper limit of their measured motor acuity, i.e., when they adopt the optimal strategy across tasks in the FSA. Faced with different target sizes, players who are experienced and skilled are predicted to flexibly adjust their behavior to maximize performance. Shot speed and precision reveal their flicking skill, and the relative skill of individual players can be objectively compared by using this motor acuity metric.

Gamers are highly invested in optimizing their performance, including factors distinct from improving FPS skill *per se*. To that end, it is of great value for this community to establish objective benchmarks for how any given influence on performance can be altered or shaped to maximize gaming outcomes. Using the FSA, the industry could readily assess within-participant differences in motor acuity to reveal the influence or relative efficacy of particular hardware (e.g., mouse, mouse pad, monitor rendering latency), software (e.g., mouse settings, axes inversion), and various other factors related to the health and physical attributes of players (e.g., posture, exercise, sleep, diet, and dietary supplements). As the scientific study of gaming performance is in its infancy, the FSA is an unprecedented and broadly applicable tool to efficiently establish foundational knowledge in the field.

The FSA not only allows for comparisons to the population, but additionally provides feedback on an individual level. That is, each individual user may measure their own baseline during an initial session of the FSA so that repeated gameplay can monitor changes relative to this baseline. This is particularly salient for esports professionals, given that currently available metrics are unsubstantiated and team performance often clouds the details of individual performance (Voida et al., 2010). Elucidating the comparable strengths and weaknesses of each member of any given esports team would not only provide a basis for coaching, but for team composition as well.

This flexible tool is also a promising training regimen for improving FPS skill. As esports professionals, the participants involved in this study were able to recognize and adapt to the incentives for SAT in both tasks as expected. Players of a lower skill or experience level would be more likely to fail at adjusting their shot behavior according to task demands, causing performance to suffer. The FSA can readily identify such faulty or insufficient adjustments in strategy. To be specific, if the slope of the line fitted to a player's shot speed by shot error is either negative, near zero, or near vertical, this indicates that the player did not make the trade-off between accuracy and speed to maximize performance between scenarios. An assessment or training protocol based on the FSA would be able to automatically provide actionable feedback: explaining the difference in incentives between the tasks based on SAT, and, for example, instructing the player to slow down or speed up depending on target size. Ultimately, repeated participation in the FSA could facilitate the improvement of flicking through experience and practice in conjunction with explicit instruction on the optimal strategies a player should use during play.

The capacity of the FSA to provide, over a short period of time with no ceiling or floor effect, a measure of motor acuity independent of strategy and SAT makes it a promising tool for a wide range of both basic and applied research; for example, visuomotor psychophysics and rehabilitation. As noted above (see Introduction), there is a paucity of academic studies examining motor acuity. The FSA has the potential to fill this critical gap in the study of human motor behavior and visuomotor psychopysics. As with any other instance of motor skill learning that is characterized by reinforcement learning [1], physical rehabilitation aims to improve motor acuity. At the same time, rehabilitation must also assess performance to provide motivating feedback for the patient and clinically relevant data for the therapist to guide rehabilitation.

Neuroplasticity drives motor learning, which itself depends on movement repetition and intensity (Nudo and Milliken, 1996; Nudo et al., 1996a,b). Neuroplasticity is also facilitated by active task engagement and enjoyment (Maclean et al., 2000, 2002; Plautz et al., 2000; Burdea, 2003; Burke et al., 2009a,b; Putrino et al., 2015; Winstein et al., 2016). Furthermore, calibrating task difficulty to an individual's skill level is critical for rehabilitation (Wolf et al., 2006), because competency is an intrinsic motivator (Przybylski et al., 2010). The FSA satisfies all of these criteria: a challenging, adaptive, and engaging task based on repetitive movement behavior. Thus, incorporating the FSA into gamified rehabilitation therapy would enable objective quantification of behavioral or motor performance (e.g., kinematics, dynamics), and could be rapidly and inexpensively deployed at scale and remotely provided to large populations.

The participants in the current study were all experienced, highly-skilled FPS players. Further investigation is required to establish the degree to which our findings generalize to the broader population of FPS players (which spans a range of skill and ability levels), the general population, or the clinical populations. Additionally, it will be crucial to establish how lower-skilled players gradually improve through training and experience, and the corresponding changes in movement kinematics (e.g., shot behavior, SAT, etc.) as well as how to optimize training.

## Data availability statement

The datasets presented in this article are not readily available because the data is proprietary. Requests to access the datasets should be directed to JL, jenny@statespacelabs.com.

## Author contributions

ID, MS, and KD carried out data analyzes and assisted with study design and manuscript preparation. DH and JL assisted with study design and manuscript preparation. WM designed and implemented the study apparatus and tasks. All authors contributed to the article and approved the submitted version.

## Conflict of interest

WM and DH are officers at Statespace Labs. ID, MS, KD, and JL are employees at Statespace Labs.

## Publisher's note

All claims expressed in this article are solely those of the authors and do not necessarily represent those of their affiliated organizations, or those of the publisher, the editors and the reviewers. Any product that may be evaluated in this article, or claim that may be made by its manufacturer, is not guaranteed or endorsed by the publisher.
