# Supplementary material for: Assessment of human expertise and movement kinematics in first-person shooter games
Source: Front Hum Neurosci. 2022 Nov 29;16:979293. doi: 10.3389/fnhum.2022.979293 (PMC9744923; doi:10.3389/fnhum.2022.979293)
Supplement: Supplementary file 1 [file Data_Sheet_1.pdf]

Supplementary Information:
Assessment of human expertise and movement kinematics in first-person shooter games

Ian Donovan, Marcia A. Saul, Kevin DeSimone,
Jennifer B. Listman, Wayne E. Mackey and David J. Heeger

Corresponding author: David J. Heeger
Email: david@statespacelabs.com

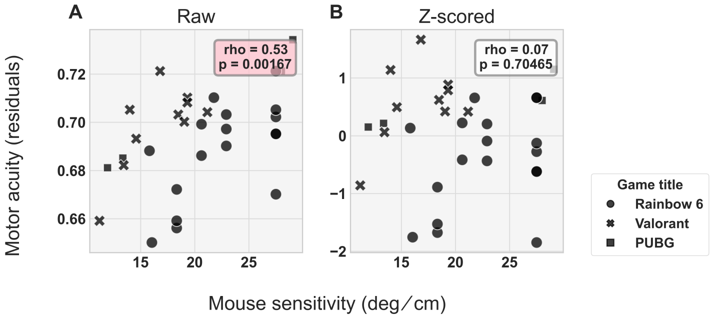

FIGURE S8

Correlation between motor acuity and mouse sensitivity. **(A)** Before regressing out sensitivity. **(B)** After regressing out sensitivity. Plot symbols, participants marked by main game titled played (see Legend). Statistical results from Spearman's Rank Correlation displayed in annotation boxes. rho = Spearman's Correlation Coefficient. p = p-value. Pink annotation boxes indicate a p-value < 0.05.

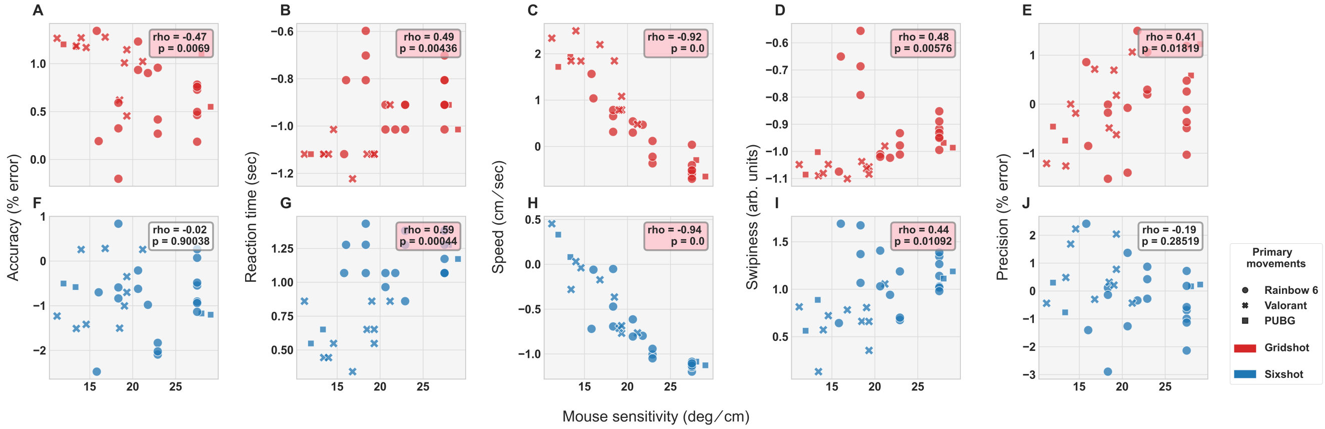

FIGURE S9

Correlation between movement kinematics (primary movements) and mouse sensitivity before regressing out sensitivity for each metric in each task. **(A-E)** Gridshot. **(F-J)** Sixshot. Plot symbols, participants marked by main game titled played (see Legend). Statistical results from Spearman's Rank Correlation displayed in annotation boxes. rho = Spearman's Correlation Coefficient. p = p-value. Pink annotation boxes indicate a p-value < 0.05.

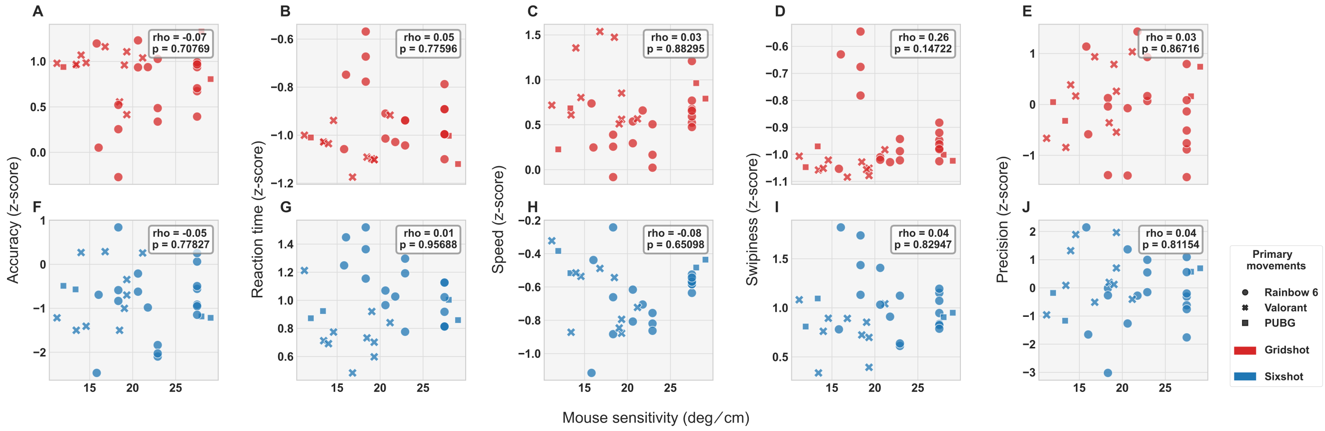

FIGURE S10

Correlation between movement kinematics (primary movements) and mouse sensitivity after regressing out sensitivity for each metric in each task. Same format as Fig. S9.

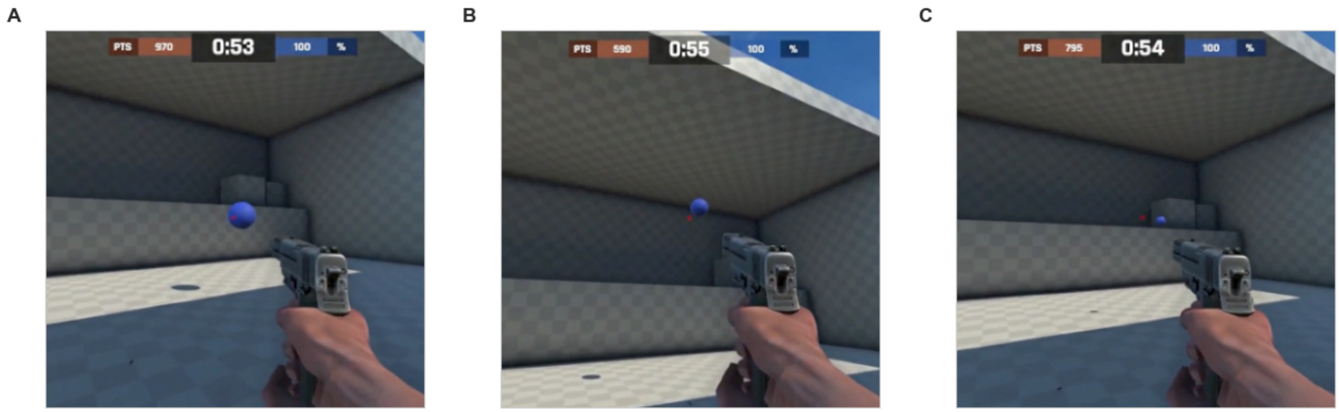

FIGURE S11

Sample screenshots of the Adaptive Reflexshot task. **(A)** Large targets. **(B)** Medium targets. **(C)** Small targets.
